# 778 Epidemiology of Food Access in an Urban Burn Population

**DOI:** 10.1093/jbcr/irad045.253

**Published:** 2023-05-15

**Authors:** Brienne Donovan, Daniel Wiese, Jingwei Wu, Kevin Henry, Lisa Rae, Jeffrey Anderson

**Affiliations:** Temple University, Philadelphia, Pennsylvania; Temple University, Philadelphia, Pennsylvania; Temple University, Philadelphia, Pennsylvania; Temple University, Philadelphia, Pennsylvania; Temple University, Philadelphia, Pennsylvania; Temple University, Philadelphia, Pennsylvania

## Abstract

**Introduction:**

Poverty is a known risk factor for burn injury and is associated with residency in food deserts and food swamps. Food deserts are areas with low or no access to nutritious, healthy foods while food swamps are areas with much higher access to high-calorie, unhealthy foods than to foods with good nutritious value. The purpose of our investigation was to determine the prevalence of residency in food deserts and food swamps in burn patients evaluated at an urban academic center and to determine the relationship between residency in these areas and the presence of patient comorbidities.

**Methods:**

We performed a retrospective chart review of patients with burn injuries seen at an ABA verified urban academic center between January 2016 and January 2022. All patients over age 18 seen in the emergency department or admitted to the burn service were included in the study. Patient residential locations were used in conjunction with the Modified Retail Food Environment Index (mREFI) data to classify residency in food deserts and food swamps. Patient demographics and comorbidities were also recorded. Chi square and ANOVA analyses were completed to determine differences between the food desert, food swamp, and good access groups.

**Results:**

A total of 3064 subjects were included in the study, of these 1370 (44.7%) were female and 1694 (55.3%) were male. In terms of access, 96 (3.1%) individuals lived in food deserts, 477 (15.6%) lived in good access areas, and 2490 (81.3%) resided in food swamps. This difference was found to be statistically significant (p < 0.0001). Age, sex, and BMI did not differ significantly between the three groups. A significantly higher percentage of food swamp residents had a diagnosis of diabetes (15.0%) as compared to subjects living in good food access areas (11.7%) and food deserts (8.3%), (p < 0.043). Hypertension was also found to be more prevalent in food swamps (24.8%) than food deserts (20.8%) and good access areas (19.3%), (p < 0.028). Finally, smoking was more common in food swamps (45.3%) than good access areas (40.4%) and food deserts (27.9%), (p < 0.0028).

**Conclusions:**

A majority of burn patients reside in food swamps and residency in these areas is associated with a higher prevalence of hypertension, diabetes, and tobacco smoking. This is the first study to investigate the epidemiology of food access in an urban burn population.

**Applicability of Research to Practice:**

Nutrition is known to be important to overall health and to wound healing in the setting of an acute injury. Our data contributes to the current literature on the role of nutrition in burns.

